# Q&A: Evolutionary capacitance

**DOI:** 10.1186/1741-7007-11-103

**Published:** 2013-09-30

**Authors:** Joanna Masel

**Affiliations:** 1 Ecology and Evolutionary Biology, University of Arizona, 1041 E. Lowell St, Tucson, AZ 84721, USA

## What is an evolutionary capacitor?

Many mutations are conditionally neutral: important under some conditions, invisible at other times. Capacitors are on/off switches affecting the visibility of a particular set of conditionally neutral variants. While in the neutral state, ‘cryptic’ genetic variants can drift to high frequency; extending the electrical analogy, accumulated cryptic variants can be seen as a kind of genetic ‘charge’ [1].

## Can you give an example of what cryptic variants are and what turns their phenotypic effects on?

Yes. When yeast ribosomes reach a stop codon, the protein Sup35 helps terminate translation. The Sup35 protein can switch between two conformations, which share an identical amino acid sequence. The normal form is soluble, while the [PSI+] prion form sucks Sup35 up into aggregates. With less soluble Sup35 available, translation can occasionally continue beyond the stop codon, so that extra amino acids are added onto the growing protein’s carboxyl terminus. The nucleotides beyond the stop codon are ‘cryptic’ because they were there all along, but normally their precise identity doesn’t matter. They are only translated into amino acids when the capacitor [PSI+] is present. Capacitance, in this case, is the consequence of a yeast lineage being able to switch between [PSI+] and [psi-].

## Isn’t it really bad for the yeast to read through stop codons?

Yeast tolerate [PSI+] remarkably well, and not just under lab conditions: the prion is seen in the wild, too [2]. Even when [PSI+] is present, most proteins still terminate normally. But [PSI+] does make a big difference to the phenotype, depending on exactly which cryptic variants are present in a given strain [2,3]. [PSI+] is relatively rare in wild yeast strains, so most of the time it is probably bad for yeast [4]. But [PSI+] increases variation, so it might have a positive effect some of the time. On those occasions, [PSI+] can smooth the path to adaptation. By providing this smoothing, evolutionary capacitors act as adaptive devices or ‘widgets’ [5] that increase evolvability, defined as the rate of appearance of heritable and potentially adaptive phenotypic variants [6].

## Isn’t that a pretty extraordinary claim in evolutionary biology?

Actually, the logic and math of the evolution of a capacitance switch is identical to that of the well-accepted case of bet-hedging [7]. The seeds of annual plants don’t all germinate straight away. If they did, and it was a bad year, the plant lineage would get wiped out. It’s better for a plant to pay a cost to hedge its bets, and have some seeds germinate while others lie dormant. Dormancy is a bet on a bad season; evolutionary capacitor switching is a bet on finding adaptive cryptic variants in a new environment.

## So the switching is completely random?

We don’t understand exactly how switching happens, but we do know that the probability of switching goes up when the yeast is under stress [8]. When everything is going well, increasing variation will be bad both for the average yeast cell, and for the population as a whole. But in a new and stressful environment, there’s a better chance that change introduces an adaptation into the population.

## How is the new phenotype inherited?

At first, the new phenotype is only present when [PSI+] is present. Prions like [PSI+] are inherited epigenetically, via the cytoplasm [9]. In a phenomenon known as ‘genetic assimilation’, a trait that was originally non-genetic can become genetic after some generations of selection [10]. As explained earlier, even in the presence of [PSI+], any given stop codon is not always read through to express the cryptic variant; this partial nature of the readthrough presumably weakens the variant’s phenotypic effects. Selection can favor changes, such as mutations in stop codons [11], that both increase the expression level of the adaptive variant, and cause it to lose its initial dependency on the presence of [PSI+] [12]. Then the prion can disappear again. The [PSI+] prion can help the yeast lineage survive long enough to find these genetic assimilation mutations [6], or it can simply help the lineage get to the final [psi-] adaptation faster through a more efficient adaptive path [12]. Unlike the case of mutator alleles, or a mutation partially knocking out Sup35 function, there is no long-term cost involved when capacitance increases the rate of adaptation [13]. The prion acts as a stopgap, finding an adaptive variant quickly, allowing its short-term inheritance, and disappearing again once the phenotype is more stably assimilated into the DNA. Lineages that are able to switch in and out of the [PSI+] state have an advantage over those that can’t, even if the [PSI+] state itself carries a cost [4].

## The [PSI+] prion is pretty weird and obscure. Can you give a more general example?

The most famous example is loss of function of the chaperone Hsp90 [14,15]. Hsp90 stabilizes many metastable signal transducers. When an organism is in trouble, Hsp90 may have too much work to do, mimicking a partial deletion. This has unpredictable consequences, depending on which cryptic variants are present in that individual’s genetic background, both in direct Hsp90 client proteins that may destabilize in the absence of Hsp90, and in genes downstream of the clients in signal transduction pathways.

## For how many generations does Hsp90 stay impaired?

That’s a good question. For Hsp90 or other evolutionary capacitors to have a significant impact on evolvability, cryptic variants need to stay switched on long enough for genetic assimilation to take place [12,16]. We don’t know if this is the case for Hsp90-mediated variation.

## Are most capacitors chaperones?

Probably not. Models of gene regulatory networks suggest that any reversible knockout - for example, as may be the result of the protein forming a prion - can act as an evolutionary capacitor [17]. Even irreversible knockouts, such as gene deletions, can facilitate adaptation [17]. Mutations to chromatin regulators [18] and other regulatory genes [19-21] reveal substantial cryptic variation. Yeast knockouts were screened for genes that provide robustness to normal developmental perturbations [22]; these genes (sometimes known as ‘phenotypic stabilizers’ rather than capacitors because they have not yet been shown to provide robustness to mutations [23]) are enriched for a number of gene types, but not chaperones. An unbiased screen of genomic regional deletions in *Drosophila* found many new sites whose deletion reveals cryptic genetic variation, but Hsp90 was not among them [24]. There may be so many potential capacitors out there that rather than capacitance being a ‘special’ mechanism, cryptic standing (or ‘crouching’) genetic variation may make routine contributions to adaptation [25].

## So capacitor genes provide mutational robustness, which is lost in the knockout?

**Figure 1.**
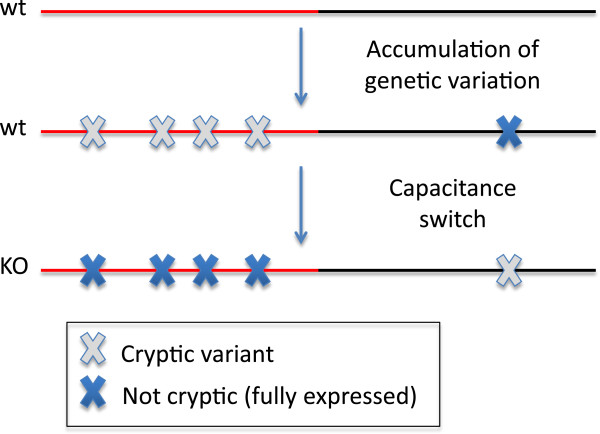
**In the red section of the genome (left), the gene acts as a capacitor, allowing cryptic genetic variation to accumulate.** In the black section of the genome, variation is fully expressed, and most is purged. When the gene is knocked out, abundant cryptic genetic variation is revealed, and a smaller amount of previously expressed variation is made cryptic. The red and black sections are shown as equally large, indicating that the wild type is not necessarily more robust to new mutations, on average, than the knockout [21].

The phenotypes of mutants such as gene knockouts are more variable than the phenotypes of the wild type [26], but this does not necessarily reflect mutational robustness. It does demonstrate the high robustness provided by the gene to the microenvironmental perturbations inherent in normal developmental processes. Robustness to mutations is more complicated, as shown in Figure 1. In the capacitance story, a knockout reveals genetic variants, showing that the intact gene provided robustness to those variants. But the knockout can also hide variants that would otherwise matter, in which case the gene is called a potentiator rather than a capacitor [27,28]. Considering both effects together, knockouts may be no less robust to mutations than wild types are [21]. But even when a gene does not increase robustness to mutations overall, it will still make some specific mutations cryptic, allowing them to accumulate until the capacitor discharges [29]. In other words, capacitors are best defined as genes with many epistatic interactions (in the classical genetic sense in which an allele at one locus masks the effect of a polymorphism at another), rather than as genes that increase mutational robustness.

## What’s so good about cryptic variation?

**Figure 2.**
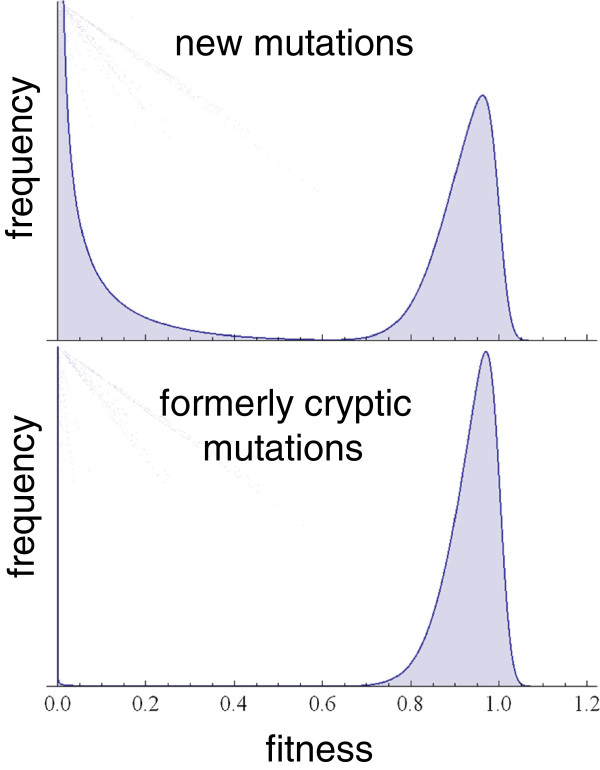
**The distribution of fitness effects of new mutations is bimodal (top).** While in a partially cryptic state, selection is strong enough to purge the left mode, while having little effect on the right mode, generating a set of cryptic variants of greatly enriched quality (bottom).

The distribution of fitness effects of new mutations is bimodal (Figure 2); most mutations either destroy something, or they tinker with it (in other words, have only small fitness effects), but their effects are rarely in-between [30]. Adaptation comes entirely from the tinkering mutations. Cryptic variants are special because they have a higher proportion of tinkering variants and fewer lethal ones. This is because there is a sharp threshold for the effectiveness of selection *s* that depends on the effective population size *N*_*e*_; when this threshold is exceeded (which is when *s* > 1/*N*_*e*_), selection works great, otherwise evolution is neutral. When genetic variants spend time in a cryptic state, they are usually expressed just a tiny bit. In other words, conditional neutrality in practice usually means conditional near-neutrality. This slight deviation from strict neutrality is enough to purge the destructive variants but retain the tinkering ones, which accumulate until the capacitor comes along to release them [31,32].

## So the advantage of cryptic genetic variation is the quality, not the quantity?

Cryptic genetic variation has both quality and quantity advantages [33]. As for quantity, capacitance helps time the release of genetic variants to coincide with episodes of stress. Because lots of variants lose their crypticity at the same time, evolution can also explore more combinations and so cross more valleys in adaptive landscapes [16,31].

## Do recessive alleles count as cryptic variants?

Sure, when an allele usually finds itself in a heterozygote, its recessive effects are cryptic to selection. In mostly asexual populations, excess heterozygosity can build up during an asexual phase, and rare episodes of sex will then act as a capacitor: outcrossing explores new allele combinations [34], while inbreeding increases phenotypic variation by converting heterozygotes to homozygotes [35]. In obligate sexuals, hybridization can have similar effects [36]. But unlike the changes brought about by a prion switch or a temporary gene downregulation, the changes brought about by sex are not so easily reversible. Sex releases cryptic variation in a single generation, but making it cryptic again is slower and more difficult. Unlike [PSI+], the capacitance switch only goes one way.
